# Proteomic Signatures of *Clostridium difficile* Stressed with Metronidazole, Vancomycin, or Fidaxomicin

**DOI:** 10.3390/cells7110213

**Published:** 2018-11-15

**Authors:** Sandra Maaß, Andreas Otto, Dirk Albrecht, Katharina Riedel, Anke Trautwein-Schult, Dörte Becher

**Affiliations:** 1 Institute of Microbiology, Department of Microbial Proteomics, University of Greifswald, Felix-Hausdorff-Str. 8, 17489 Greifswald, Germany; andreas.otto@uni-greifswald.de (A.O.); anke.trautwein-schult@uni-greifswald.de (A.T.-S.); dbecher@uni-greifswald.de (D.B.); 2 Institute of Microbiology, Department of Microbial Physiology and Molecular Biology, University of Greifswald, Felix-Hausdorff-Str. 8, 17489 Greifswald, Germany; dirk.albrecht@uni-greifswald.de (D.A.); riedela@uni-greifswald.de (K.R.)

**Keywords:** *Clostridiodes difficile*, antibiotics, proteomics, protein synthesis, 2D PAGE

## Abstract

The anaerobic pathogen *Clostridium difficile* is of growing significance for the health care system due to its increasing incidence and mortality. As *C. difficile* infection is both supported and treated by antibiotics, a deeper knowledge on how antimicrobial agents affect the physiology of this important pathogen may help to understand and prevent the development and spreading of antibiotic resistant strains. As the proteomic response of a cell to stress aims at counteracting the harmful effects of this stress, it can be expected that the pattern of a pathogen’s responses to antibiotic treatment will be dependent on the antibiotic mechanism of action. Hence, every antibiotic treatment is expected to result in a specific proteomic signature characterizing its mode of action. In the study presented here, the proteomic response of *C. difficile* 630∆*erm* to vancomycin, metronidazole, and fidaxomicin stress was investigated on the level of protein abundance and protein synthesis based on 2D PAGE. The quantification of 425 proteins of *C. difficile* allowed the deduction of proteomic signatures specific for each drug treatment. Indeed, these proteomic signatures indicate very specific cellular responses to each antibiotic with only little overlap of the responses. Whereas signature proteins for vancomycin stress fulfil various cellular functions, the proteomic signature of metronidazole stress is characterized by alterations of proteins involved in protein biosynthesis and protein degradation as well as in DNA replication, recombination, and repair. In contrast, proteins differentially expressed after fidaxomicin treatment can be assigned to amino acid biosynthesis, transcription, cell motility, and the cell envelope functions. Notably, the data provided by this study hint also at so far unknown antibiotic detoxification mechanisms.

## 1. Introduction

As a spore-forming anaerobic bacterium that primarily causes antibiotic-associated diarrhea, *Clostridium difficile* (recently reclassified from *Peptoclostridium difficile* to *Clostridiodes difficile* [1]) represents an enormous financial burden for the health care system [2]. This is even more important as the incidence in hospitals is increasing, both in frequency and severity, resulting in considerable morbidity and mortality. In clinical practice most patients experiencing a first episode of *Clostridium*-associated diarrhea are cured by treatment with metronidazole or vancomycin, the former preferred for mild or moderate episodes and the latter for severe episodes [3,4,5]. Both antibiotics exhibit different chemical structures resulting in varying mechanisms of action. The glycopeptide antibiotic vancomycin inhibits cell wall synthesis by binding to the D-Ala-D-Ala residue of the UDP-MurNAc-pentapeptide [6], an essential precursor in the peptidoglycan synthesis. For a long time, oral vancomycin was the only therapy approved to treat *C. difficile* infection (CDI), and therefore it is currently regarded as the gold standard in clinical trials. However, today vancomycin is no longer considered a first-line treatment due to the increasing incidence of vancomycin-resistant enterococci [7,8], which continue to be a clinical challenge despite the availability of new therapeutic agents [9].

The nitroimidazole antibiotic metronidazole in its partially reduced form inhibits nucleic acid synthesis by disrupting the DNA of anaerobic microbial cells. However, increasing resistance of *C. difficile* against metronidazole has been reported [10,11,12,13]. Moreover, this drug was associated with central nervous system neurotoxicity in patients [14].

Although application of either vancomycin or metronidazole usually cures CDI, 20–25% of patients have recurrent disease after the treatment is stopped [15]. Due to its limited activity against the normal gut flora and lower relapse rates, fidaxomicin is now more frequently used for CDI treatment, especially for recurrent cases [16,17]. The macrocyclic antibiotic fidaxomicin (previously known as OPT-80, PAR-101, tiacumicin B, and difimicin) binds to the DNA template-RNA polymerase complex and prevents the initial separation of DNA strands, which inhibits mRNA synthesis by hindering the RNA polymerase σ-subunit [18]. Fidaxomicin received approval in Germany and in the USA in 2011 for CDI treatment in adults. Since then, it is applied more frequently, as it provides improved sustained cure rates compared to vancomycin or metronidazole [8,19]. Indeed, fidaxomicin demonstrates only moderate inhibitory activity against the healthy colonic flora, including Gram-positive bacteria other than *C. difficile*, anaerobes, and enteric Gram-negative bacilli [20]. This may be explained by its unique target site, which differs among bacterial species [21].

As various *C. difficile* strains are already resistant to many antibiotics, particularly in the case of quinolones and the emergent ribotype 027 strains [22], it is of increasing importance to understand the detailed adaptation mechanisms of this pathogen to antibiotic treatment. In order to do this in a comprehensive manner, it is beneficial to detect not only changes in protein amounts, but also in protein synthesis. Upon cellular stress protein synthesis rates are rapidly changing in order to react to the varying conditions. Significant differences in protein synthesis can therefore be detected with high sensitivity at an early time point after antibiotic treatment. On the other hand, the protein amount always results from the extent of protein synthesis and the extent of simultaneous protein degradation. Indeed, changes in protein abundance occur much slower compared to changes in protein synthesis and are therefore harder to observe during the early phases of adaptation. Changes in protein synthesis can be observed easily by separating radioactively pulse-labeled proteins in 2D gels. Hence this technique is still the most sensitive and most widely applied approach for the analysis of protein synthesis rates [23,24,25].

In this study, the response of *C. difficile* 630∆*erm* to vancomycin, metronidazole, or fidaxomicin stress was investigated in the exponential growth phase. Therefore, protein synthesis rates and changes in protein amounts were quantified during the first 30 min after antibiotics addition. Comparative 2D PAGE-based analysis of protein patterns revealed signature proteins for each treatment, and therefore, provides new insights into antibiotic-caused physiological disturbance and potential development of antibiotic resistance.

## 2. Materials and Methods

### 2.1. Bacterial Growth and Antibiotic Stress

**Figure 1:**
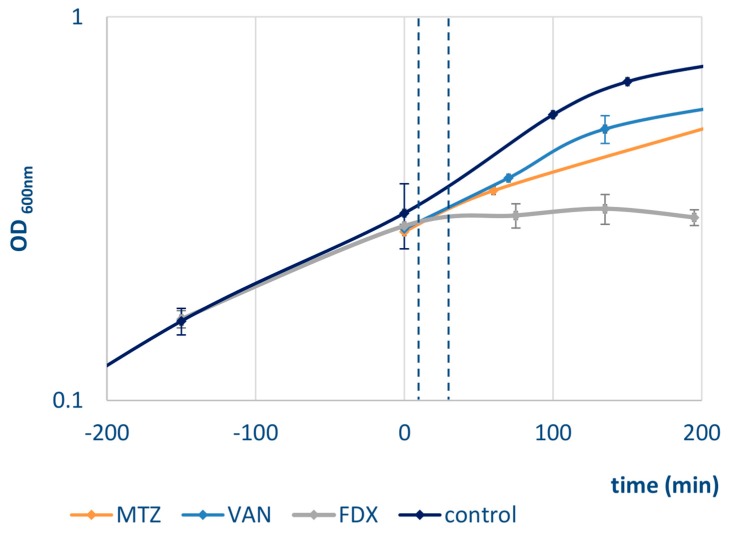
Growth of *C. difficile* 630∆*erm* was performed at 37 °C in brain heart infusion (BHI) in an anaerobic chamber. At time = 0 min (exponential growth phase, OD_600nm_ of 0.4) cultures were stressed with either metronidazole (MTZ, orange), vanomycin (VAN, light blue) or fidaxomicin (FDX, grey) or left untreated (control, dark blue). The antibiotic concentration used resulted in reduced growth rates compared to an untreated control culture. Stress samples were taken 10 min and 30 min after addition of the antibiotic (dashed lines).

*Clostridium difficile* 630*∆erm* [26] was grown at 37 °C in brain heart infusion (BHI) in an anaerobic chamber. In the exponential growth phase (OD_600nm_ of 0.4), cultures were stressed with sublethal concentrations of vancomycin (13 µg/mL), metronidazole (3.75 µg/mL), or fidaxomicin (4 ng/mL), respectively, resulting in reduced growth rates compared to an untreated control culture (Figure 1). Experiments were performed in three biological replicates.

### 2.2. Radioactive Pulse-Labeling, Cell Harvest, and Protein Preparation

Cultures were pulse-labeled for 10 min each with 150 µCi of l-[^35^S]-methionine per mL at an OD_600nm_ of 0.4 (for control), and 10 min and 30 min after antibiotic stress as described previously [27]. Briefly, l-[^35^S]-methionine incorporation was stopped by adding unlabeled methionine (100 mM) and chloramphenicol (1 mg/mL). Cells were harvested by centrifugation and were subsequently resuspended in TE-buffer containing the protease inhibitor PMSF (10 mM Tris, 5 mM EDTA (ethylenediaminetetraacetic acid), 1 mM PMSF (phenylmethylsulfonyl fluoride), pH 7.5). Cells were mechanically disrupted using the Precellys 24 homogenizator (PeqLab, Erlangen, Germany; 3 × 30 s at 6.5 m s^−1^). Protein concentrations of extracts were determined using the Bradford assay [28] and incorporated radioactivity was determined by scintillation counting.

### 2.3. 2D PAGE and Autoradiography

Isoelectric focusing was done in the pH range 4–7 with 18 cm IPG strips (SERVA, Heidelberg, Germany), loaded with 100 µg protein in rehydration solution as described elsewhere [29]. Focusing occurred in the Protean i12 IEF Cell (Bio-Rad, München, Germany) in a five-stepped voltage gradient (150 V until 150 Vh, 300 V until 300 Vh, 600 V until 600 Vh, 1500 V until 1500 Vh, and 3000 V until 57.5 kVh had been reached). The SDS PAGE was done in the horizontal system HPE Tower (SERVA) using precast gels (2D HPE Large Gel NF 12.5%, SERVA). Gels were stained with LavaPurple (SERVA) according to the manufacturer’s instructions. Stained protein spots were detected with a Typhoon 9400 multi-mode imager (GE Healthcare Europe, Frankfurt, Germany) by excitation with the green laser (532 nm) and detection with a 560 nm long-pass filter. Subsequently, gels were dried on a chromatography paper backing by using a heated vacuum dryer. For autoradiography of the radioactively labeled protein pattern, dried gels were exposed to storage phosphor screens (Molecular Dynamics Storage Phosphor Screen, 20 by 25 cm) for a time span corresponding to the amount of radioactivity separated on the gel and ensuring usage of the whole dynamic range by the strongest spot. Screens were scanned using a Typhoon 9400 multi-mode imager at a resolution of 200 μm by excitation with a red laser (633 nm) and detection with a 390 nm band-pass filter.

### 2.4. Processing of Gel Images and Statistical Analysis

Gel images were analyzed employing Delta2D 4.6.3 software (Decodon GmbH, Greifswald, Germany). Normalized spot volumes were exported from the software and the z-score transformed before running an ANOVA-test for each antibiotic analyzed (control vs. 10 min stress vs. 30 min stress). Spots and proteins with significantly changed amount exhibited a *p*-value < 0.05 and an average log_2_ fold change >|0.8|.

### 2.5. Spot Identification Using MALDI-TOF-MS

All spots visualized by the fluorescent stain were selected for identification with MALDI-TOF-MS according to previously published protocols [30]. Briefly, protein spots were excised from stained gels using an Ettan spot picker (Amersham Biosciences, Freiburg, Germany) and spots of similar staining intensity were sorted into 96-well microplates Greiner Bio-One (Greiner, Frickenhausen, Germany). Tryptic digest with subsequent spotting onto a MALDI target was carried out automatically with the Ettan Spot Handling Workstation (Amersham Biosciences). Gel pieces were washed, dried, and digested using trypsin. The concentration of trypsin was adjusted to the intensity of protein spots in order to ensure that the protease-to-protein concentration was in an acceptable range for proteins of differing abundances. Peptides were extracted from the gel, transferred into a new microplate, and dried completely. Dried peptides were resuspended in a matrix (α-cyano-4-hydroxycinnamic acid) and spotted onto the MALDI target. Samples were allowed to dry on target at room temperature before measurement. Analysis by MALDI-TOF was carried out on an AB SCIEX TOF/TOF 5800 Analyzer (AB Sciex, Darmstadt, Germany). Spectra were recorded in a mass range from 900 to 3700 Da with a focus mass of 1700 Da. For one main spectrum, 25 sub-spectra with 100 shots per sub-spectrum were accumulated using a random search pattern. If the autoproteolytic fragment of trypsin with the mono-isotopic (M + H)^+^ *m*/*z* at 2211.104 reached a S/N of at least 40, an internal calibration was automatically performed as one-point-calibration using this peak. The standard mass deviation was below 0.15 Da. Only if the automatic mode failed (in less than 1%), calibration was carried out manually. The five most intense peaks from the MS spectra were selected for MS/MS analysis. To acquire a dependent MS/MS scan, 20 sub-spectra with 125 shots per sub-spectrum were accumulated using a random search pattern. The internal calibration was automatically performed as one-point-calibration with the mono-isotopic arginine (M + H)^+^ m/z at 175.119 or lysine (M + H)^+^ *m*/*z* at 147.107 if these peaks reached a S/N of at least 5. Peak lists were created using GPS Explorer Software Version 3.6 (build 332) with the following settings: 900–3700 Da mass range, 20 peaks per 200 Da peak density, minimal S/N of 15 and a maximum of 65 peaks per spot. For MS/MS settings, a mass ranged from 60 Da to reduced mass of precursor (−20 Da), a peak density of 50 peaks per 200 Da and maximal 65 peaks per precursor were used. Peak lists were created for a minimal S/N of 10. All peak lists were analyzed using the Mascot search engine version 2.4.1 (Matrix Science Ltd., London, UK) with a specific database for *C. difficile* 630Δ*erm* [31]. Identifications were considered as correct if the protein score was >51.

## 3. Results

In this study, a method for radioactive protein labeling with ^35^S in *C. difficile* was developed. Moreover, comparative 2D PAGE was applied for the first time to detect, visualize, and quantify protein amounts and protein synthesis rates during stress adaptation in *C. difficile*. This approach was used to separate proteins synthesized 10 and 30 min after antibiotic stress in the laboratory strain *C. difficile* 630*∆erm*. Adaptation to antibiotics was monitored after adding sublethal concentrations of vancomycin, metronidazole, or fidaxomicin to exponentially growing cells, resulting in reduced growth rates compared to an untreated control culture (Figure 1). Fluorescence staining of proteins additionally allowed for quantification of protein amounts actually present in the samples. Proteins with a pI of 4 to 7 were separated on 2D gels, resulting in the detection of 1167 spots with assigned protein identification. This led to the quantification of 425 proteins in all conditions examined, of which 107 proteins were significantly altered in either amount or synthesis after treatment with at least one of the antibiotics tested (Supplementary Tables S1 and S2).

Essential proteins represent possible targets of antimicrobial drugs. Until now, a list of putative essential proteins in *C. difficile* has only been published for strain R20291, which comprises 377 proteins [32]. Hence, homologous genes in *C. difficile* 630*∆erm* were identified using Proteinortho [33] and their protein amount and synthesis rate were checked in the dataset provided in this study. Indeed, 103 homologous proteins could be quantified of which 20 were significantly altered in either synthesis or protein amount. Among all differentially regulated proteins ribosomal proteins, tRNA ligases and enzymes involved in cell wall synthesis could be identified (Table 1). Those regulated essential proteins may represent (direct or indirect) targets of the antibiotics examined which are affected independently and regardless of the primary mode of action of those antimicrobial compounds.

### 3.1. Proteomic Signatures for Vancomycin, Metronidazole, and Fidaxomicin Stress

**Figure 2:**
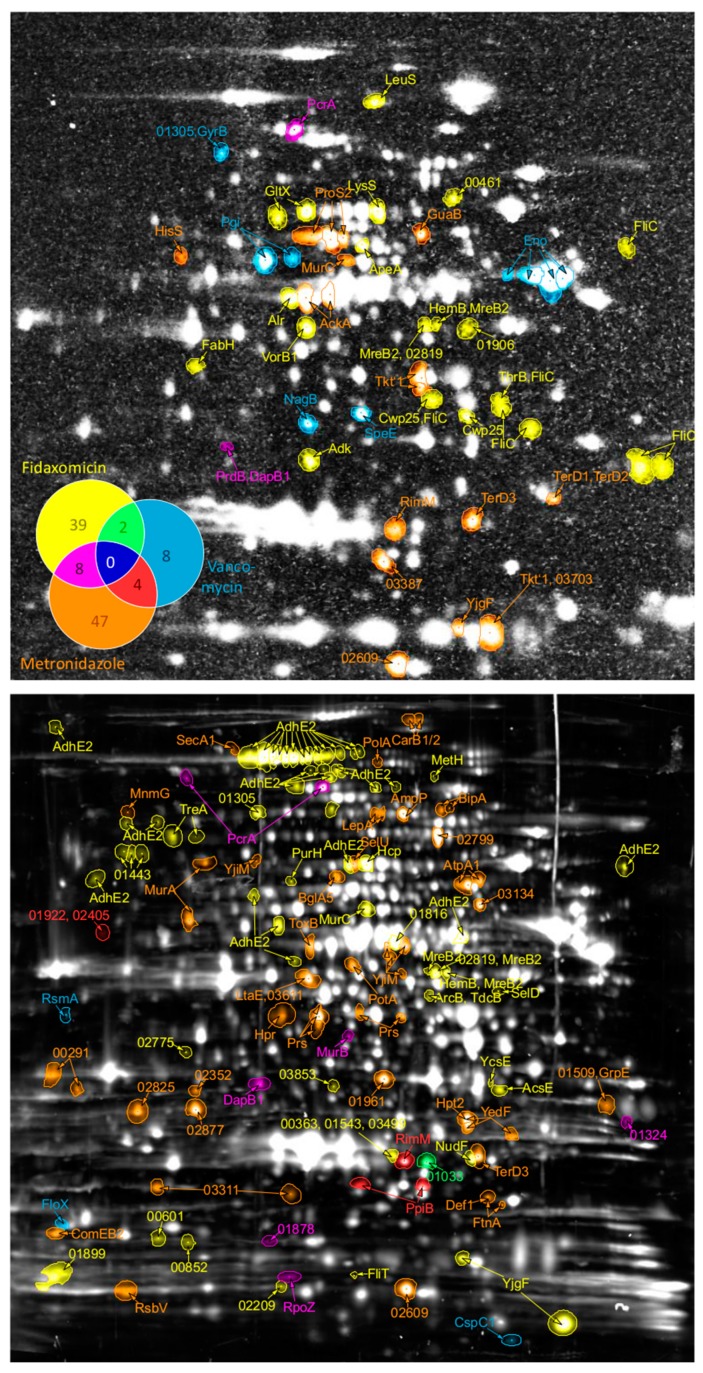
Protein pattern of antibiotic signatures in *C. difficile*. Spots of significantly changed proteins are indicated on the autoradiogram (upper part) for proteins with altered protein synthesis rate or on the 2D gel stained for total protein amount (lower part) for proteins with significantly altered amounts. Protein names (if available) or accession numbers (excluding “CDIF630erm_”) according to Dannheim et al. [31] were used as labels. The colors as assigned in the Venn-diagram (top-left) indicate after which antibiotic treatment the proteomic response became detectable. Numbers in the Venn-diagram represent the quantity of proteins altered in synthesis rate or abundance after antibiotic treatment.

Treatment of bacterial cells with antibiotics causes physiological responses aimed at elimination of cellular damage and prevention of further impairment by the drug. Thereby the pattern of cellular responses is highly dependent on the antibiotic target and mechanism of action [34]. Hence, every antibiotic treatment will result in a proteomic signature indicating the mode of action of this drug. In this study, separation of proteins by 2D PAGE did not only allow to quantify changes in protein synthesis and accumulated protein amounts but also supports the deduction and visualization of proteomic signatures during treatment of *C. difficile* with vancomycin, metronidazole, or fidaxomicin (Figure 2). The results indicate very specific cellular responses to the antibiotics, as more than 87% of the differentially expressed proteins exhibit changes only after treatment with one of the antimicrobial compounds. This results in proteomic signatures, which are very specific for each drug. In detail, eight signature proteins could be deduced for vancomycin stress, and 47 and 39 signature proteins were detected for metronidazole and fidaxomicin treatment, respectively (Table 2). However, a small number of proteins were regulated by more than one antimicrobial compound. Specifically, the responses to metronidazole and fidaxomicin showed more similarities when compared to each other than when compared to vancomycin stress (Figure 2). Indeed, along with the reduced growth rate caused by treatment with both antimicrobials, fidaxomicin and metronidazole, altered amounts of proteins involved in DNA metabolism, transcription or cell wall metabolism were detected.

.

### 3.2. Proteomic Adaptation to Vancomycin

**Figure 3:**
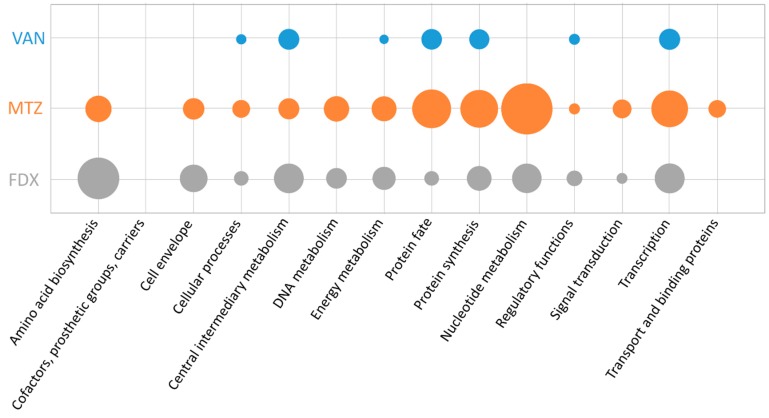
Functional categorization of significantly altered proteins according to TIGRFAMs [36]. The number of significantly altered proteins after treatment with vancomycin (VAN, blue), metronidazole (MTZ, orange), or fidaxomicin (FDX, grey) correlated to the total number of proteins in the respective functional category are represented by the circle’s size. Hence, enriched categories are represented by bigger circles.

The proposed mechanism of action for vancomycin includes the binding of the antimicrobial molecule to an essential precursor in cell wall synthesis, which results in a slow killing rate of this antibiotic and causes a bacteriostatic effect in *C. difficile* [35]. In the dataset presented here, only 14 proteins were significantly altered in either synthesis or amount after treatment (Supplementary Tables S1 and S2), of which eight proteins represent signature proteins for vancomycin (Table 2). Changes in synthesis rate or abundance could be detected for proteins involved in intermediary metabolism as well as in the regulation of other proteins’ synthesis or fate (according to TIGRFAMs [36], Figure 3). However, the number of differentially expressed proteins during vancomycin stress is strikingly lower compared to the number of proteins altered after treatment with metronidazole or fidaxomicin, respectively.

Signature proteins for vancomycin stress fulfil various cellular functions, suggesting that the differential expression of those proteins in the early adaptation phase is rather a secondary than a direct effect of vancomycin treatment. However, this study still revealed signature proteins directly related to the mode of action of vancomycin, namely the glucosamine-6-phosphate deaminase (CDIF630erm_01147), the glucose-6-phosphate isomerase Pgi (CDIF630erm_03585), both providing precursors for cell wall biosynthesis and the peptidyl-prolyl *cis*-*trans* isomerase B PpiB (CDIF630erm_00459) involved in protein homeostasis of the cell envelope.

### 3.3. Proteomic Adaptation to Metronidazole

The antimicrobial function of metronidazole is mediated by the disruption of DNA in anaerobic cells that results in inhibition of nucleic acid synthesis. In the study presented here, 59 proteins of *C. difficile* were significantly altered in either synthesis or amount after metronidazole stress (Supplementary Tables S1 and S2). A significant fraction of those proteins fulfil functions in intermediary, energy, or nucleotide metabolism or are assigned to the functional categories “protein synthesis” and “protein fate”. Moreover, several proteins with changed amount or regulated synthesis after metronidazole stress play a role in amino acid metabolism, transcription or cellular processes including adaptation to atypical conditions (Figure 3).

Due to the proposed mode of action of metronidazole, it could be expected to find differentially regulated proteins involved in DNA replication, recombination, and repair. Indeed, the deoxycytidylate deaminase ComEB2 (CDIF630erm_03788), DNA helicase PcrA (CDIF630erm_00456), DNA polymerase I PolA (CDIF630erm_01275), and carbamoyl-phosphate synthase CarB (CarB1 (CDIF630erm_03910), CarB2 (CDIF630erm_03912), identified in the same spot on the 2D gel) were significantly more abundant after treatment with metronidazole.

Additionally, the proteomic signature of metronidazole stress is characterized by alterations of proteins involved in protein biosynthesis and protein fate pointing to a substantial reprogramming of protein expression during adaptation to metronidazole. In this context it should be noted that 13 tRNA ligases (AlaS, AsnC, GlnS, GltX, GlyQ, GlyS, HisS, IleS LeuS, LysS, MetG, ThrS, ValS) were found to be increased more than 1.75-fold in either protein amount or protein synthesis during adaptation. The energy necessary for the active reorganization of the proteome is provided by the activation of the energy metabolism in the early adaptation phase as supported by the detection of differentially expressed proteins assigned to this functional category.

The specificity of metronidazole against anaerobic microbes is caused by nitroreductases and oxidoreductases that are present in these bacteria. Both enzyme classes activate metronidazole via reduction resulting in unstable and/or less reduced intermediates that damage DNA by strand breakage, helix destabilization, and helix unwinding leading to cell death [37]. Although 43 nitroreductases or oxidoreductases have been annotated in the *C. difficile* genome, only ten of those enzymes could be quantified in this study and none of them were significantly altered in either protein synthesis or amount.

Furthermore, it is worth mentioning that a significantly increased protein synthesis was detected for the tellurium resistance protein TerD, putatively involved in detoxification of the antimicrobial. The tellurium resistance protein is represented by its paralogues TerD1 (CDIF630erm_01811) and TerD4 (CDIF630erm_01998) 30 min after addition of metronidazole to the *C. difficile* culture. However, although no increase in protein amount was detectable during the early phase of adaptation, changes in the protein amount of TerD in later time points after metronidazole stress can be expected based on protein synthesis data.

### 3.4. Proteomic Adaptation to Fidaxomicin

Fidaxomicin functions by binding to the DNA strand-RNA polymerase-complex, which prevents successful mRNA synthesis. In the current study 49 proteins where significantly altered in either amount or synthesis rate after treatment with fidaxomicin (Supplementary Tables S1 and S2). Thereby differentially regulated proteins can be assigned to a variety of biological functions, of which amino acid and protein biosynthesis, transcription, nucleotide metabolism, cell motility and functions in the cell envelope are most prominent (Figure 3). More specifically, ArcB (CDIF630erm_00657), DapB1 (CDIF630erm_03522), MetH (CDIF630erm_03918), TdcB (CDIF630erm_02763), and ThrB (CDIF630erm_02348), involved in synthesis of aspartate, glutamate, methionine, threonine, or serine, respectively, have been found to be upregulated in response to fidaxomicin.

Due to the mode of action of fidaxomicin, it can be expected that proteins with functions in transcription and translation are regulated when cells are facing this drug. This hypothesis can be supported by the data presented here. While YigF (CDIF630erm_02762), involved in RNA degradation, is downregulated, other proteins with functions during transcription and translation, like RpoZ (CDIF630erm_02841), or various tRNA ligases (LeuS (CDIF630erm_02772), LysS (CDIF630erm_03872), GltX (CDIF630erm_00114)) were found to be significantly upregulated.

Moreover, the current study suggests that treatment of *C. difficile* with fidaxomicin alters processes in the cell envelope. In more detail, Alr (CDIF630erm_03774), Cwp25 (CDIF630erm_00963), MreB2 (CDIF630erm_01292), MurB and MurC (CDIF630erm_03707 and CDIF630erm_03832) were differentially expressed during fidaxomicin stress.

Additionally, it should be mentioned that treatment of *C. difficile* with fidaxomicin alters the amount of two transcriptional regulators that have not been further characterized. The regulatory protein of the GntR-family (CDIF630erm_01543) was found with increased amounts as soon as 10 minutes after application of fidaxomicin to the bacterial culture. In contrast, the transcriptional regulator of the MarR-family (CDIF630erm_00601) was detected with lowered amounts during treatment.

## 4. Discussion

### 4.1. Technical Aspects of Gel-Based Approaches to Derive Antibiotic Signature

Due to the increasing incidence of antibiotic-resistant *C. difficile* it is of great importance to understand the detailed adaptation mechanisms of this widespread pathogen to antibiotic treatment. In this context the analysis of proteins is of great interest as these molecules catalyze crucial metabolic reactions as well as cellular processes necessary during pathogenesis. Indeed, it is the proteome which translates the genome sequence of an organism into cellular functions and structures, and therefore proteins can be regarded as the main players of life. Therefore, proteomics allows to monitor cellular adaptation to changing environmental conditions, which may also give hints to the mechanism of action of different antibiotics. In order to investigate the proteomic adaptation of *C. difficile* to commonly used antibiotics in a comprehensive and sensitive manner, it is beneficial to detect not only changes in protein amount, but also in protein synthesis. The availability of quantitative data on protein amount and protein synthesis in this study enabled the sensitive detection of alterations in TerD synthesis after metronidazole stress. Indeed, the induced synthesis of the putatively detoxifying enzymes TerD1 and TerD4 could be detected already during early time points of adaptation, whereas it remains elusive if detectable changes in protein amounts can be observed later after antibiotic stress. An example of limitation for a study combining investigations on both protein synthesis rate and protein amount is AdhE2 (CDIF630erm_03250), of which increased amounts could be detected after fidaxomicin stress. However, the fact that no radioactive signal could be detected for this spot hints to an insufficient labeling of the newly synthesized AdhE2.

The preferred and still most widely used technique for visualization and quantification of protein synthesis is pulse-labeling with radioactive amino acids [23,24,25]. However, a prerequisite for successful labeling of newly synthesized proteins is the sufficient incorporation of the radioactive label in the proteins. Labeling with ^35^S works best, if neither methionine nor any other unlabeled sulfur source is present in the medium during growth. Moreover, labeling with radioactive methionine is most efficient if all methionine offered is incorporated into the proteins synthesized during the investigated time span. However, both methionine and cysteine are required for *C. difficile* growth in any medium and both amino acids are not only used for protein biosynthesis but also for energy metabolism [38]. Therefore, radioactive labeling efficiencies had to be evaluated and the protocols for pulse-labeling of *C. difficile* had to be adapted. The modified method applied in this study opens the possibility to investigate changes in protein synthesis in *C. difficile* with radioactive pulse-labeling. One disadvantage, however, is the need for doses of radioactivity to label sufficient protein amounts for a single 2D gel ten times higher than for comparable experiments in *Bacillus subtilis* [39]. In order to correct for proteomic effects that are caused by damage to the DNA, which might be introduced by the high amount of radioactivity, the control cultures have also been labeled with ^35^S-methionine. Hence, all differences in either protein amount or protein synthesis rate described in this study are solely caused by the antibiotic treatment.

In order to be able to compare protein adaptation after treatment with several antimicrobial agents it is necessary to select appropriate sampling points. However, if the mode of action of the antibiotics examined differs significantly it is possible that the time span necessary to detect a proteomic or even a phenotypic adaptation varies. In this study, the number of differentially regulated proteins during vancomycin stress is strikingly lower compared to the number of proteins altered after treatment with metronidazole or fidaxomicin. Although the sampling points selected for the experiments seem to be suitable for metronidazole and fidaxomicin stress, they might be too early after vancomycin stress to detect proteomic changes. This idea is supported by the fact that for none of the proteins exhibiting differential protein synthesis rates after treatment with vancomycin changes in protein amount could be detected. Moreover, in a study on vancomycin stress in *Staphylococcus aureus* the authors only took samples 100 min after adding vancomycin to the bacterial culture in order to detect adaptive changes in protein amount [40]. In this study, growth inhibitory effects became visible only after 30 min of treatment. Indeed, the delayed response to the antibiotic was also observed in *C. difficile*, where reduction in growth rate started 60 min after addition of vancomycin. However, the current study aimed not only at monitoring changes in protein amount but also at examining the proteomic adaptation on the level of protein synthesis, which is usually an immediate cellular response to perturbations. In the case of metronidazole and fidaxomicin stress, sampling after 60 min of treatment might be too late to monitor the early proteomic responses to those antibiotics. Hence, the selection of early sampling points was reasonable as changes in protein synthesis within the first 30 min of stress were expected for all three antibiotics.

Additionally, the study on *S. aureus* during vancomycin treatment revealed the most striking impact on the amount of proteins localized in or attached to the cell wall [40]. However, those proteins cannot be covered in a 2D gel-based study, as the quantification of this protein class requires specific protein enrichment and sample preparation workflows that are incompatible with 2D PAGE. The same holds true for a more detailed analysis of adaptational processes in the cell envelope after treatment with fidaxomicin. Hence, it remains speculative if the putative reorganization of cell surface components results from attempts to repel the antimicrobial compound or if the data hint to independent or even secondary effects of fidaxomicin treatment. Further research may provide more details on the impact of fidaxomicin and vancomycin to the cell envelope of *C. difficile*. Moreover, proteins associated to the bacterial cell surface or secreted into the environment represent important virulence factors and may also be directly involved in the recognition, targeting, and detoxification of antimicrobials. Hence, it may also be useful to complement the available data sets in follow-up studies with comprehensive views on other subcellular proteome fractions than the soluble proteins in the cell.

### 4.2. Protein Signatures in Antibiotic Research in C. difficile

A vast collection of comprehensive knowledge on bacterial adaptation to antibiotic stress is a prerequisite to understand the complex interaction of bacteria, drugs, and environment. This is even more important in pathogenic bacteria where this information is necessary to discover activities that reduce the development of resistance or prevent resistant strains from spreading. However, most data available focus on well described model organisms like *Escherichia coli*, *B. subtilis*, or *S. aureus*.

The largest publicly available compendium to date contains proteome profiles of the Gram-positive model organism *B. subtilis*. These data are, like in this study, based on 2D PAGE, and contain proteome patterns of *B. subtilis* treated with 30 different antimicrobial compounds [25]. Such comprehensive datasets enabled the authors to group the proteomic adaptation according to the antibiotics examined. Interestingly, the grouping of antibiotics based on the previously described responses of *E. coli* [41] was the same as the grouping based on the responses of *B. subtilis*, while the marker (responder) proteins were species-specific [34]. This may also hold true in other bacteria such as *C. difficile*. Hence, the compilation of *C. difficile* proteomic signatures in this and following studies will facilitate a successful grouping of different physiological strategies to counteract antibiotics, which will subsequently allow for the deduction of the mechanism of action of new antibiotics aiming at treating CDI.

In addition to the opportunity to group the proteomics adaptation to antibiotic treatments according to the (putative) mode of action, the deduction of signature proteins enhances the knowledge on resistance development and spreading as it defines specific responses to antimicrobial compounds. Indeed, the expression of only a little number of proteins is sufficient to render a microorganism resistant to a particular antibiotic. Such proteins function as efflux pumps, antibiotic-degrading or modifying enzymes, or represent a mutated antibiotic target. Most often, the antibiotic-regulated proteins can be linked directly or indirectly to the physiological state of the bacterial cell during antibiotic treatment. Hence, it was surprising that only very few signature proteins identified in this study seem to be directly linked to the drug applied. However, proteins regulated after antibiotic treatment may not only be directly involved in compensating for the loss of a particular function or in repairing the damage inflicted by an antibiotic but could also be indirectly connected to the anticipated mechanism of action of a specific antimicrobial. Moreover, the unique regulation of signature proteins in response to a particular antibiotic may point to unexpected targets that may also be prone to variations during resistance development.

### 4.3. Response to Metronidazole in Other Anaerobic Bacteria

Previous investigations on the adaptation of *C. difficile* NAP1, *Bacterioides fragilis*, and *Helicobacter pylori* to metronidazole revealed the involvement of various protein groups in the detoxification of metronidazole and the development of resistance [42,43,44,45]. Those proteins are involved in DNA replication, recombination and repair, protein synthesis and regulation of protein stability, or represent stress proteins, or nitroreductases and oxidoreductases.

In a microarray analysis of the transcriptional responses of *C. difficile* grown in the presence of a sub-inhibitory concentration of metronidazole, it has been reported that the genes coding for ribosomal proteins, subunits of the RNA polymerase, elongation and translation factors as well as the majority of tRNA ligases were coordinately upregulated [43]. Although the upregulation of tRNA ligases can also be observed in *C. difficile*, the influence of metronidazole treatment on other proteins involved in protein synthesis was not that clear. In more detail, five proteins of the large ribosomal subunit and six proteins of the small ribosomal subunit, as well as three subunits of the RNA polymerase (RpoA, RpoB, RpoZ) have been quantified in this proteomics study. However, none of those proteins was significantly upregulated.

Metronidazole resistance in *B. fragilis* and *H. pylori* involves genes encoding nitroreductases and oxidoreductases [46,47]. These enzymes activate metronidazole leading to DNA damage and ultimately to cell death [37]. However, changing the abundance of nitroreductases and oxidoreductases might not be the first strategy of *C. difficile* to adapt to metronidazole. Still, the alteration of enzyme activity by adaptation of protein amount, post-translational protein modification or even mutations in their encoding genes, as described earlier for *B. fragilis* and *H. pylori* may be viable during long-term adaptation to metronidazole.

In summary, although differences in the species examined and the experimental conditions of the different studies are obvious, the already published effects are in accordance with the data of the current study.

### 4.4. Detoxification of Antimicrobial Compounds in C. difficile

Besides repel, interception, and export, antimicrobial compounds can be detoxified by degradation or modification. During metronidazole stress the synthesis rate of the tellurium resistance protein TerD was found to be increased in *C. difficile*. It is known from other pathogenic bacteria that these genes mediate responses to diverse extracellular stimuli and may also be involved in detoxification of antimicrobials [48]. However, the mechanisms of detoxification by tellurium resistance proteins are still unknown [49]. In the case of metronidazole stress in *C. difficile* 630*∆erm* the synthesis of two of the five paralogues of TerD were significantly increased suggesting a function of Ter-proteins in Clostridiales during detoxification of metronidazole as well.

During adaptation to antibiotics, the alteration of transcriptional regulators in terms of amount or post-transcriptional modification possibly enables the cell to react to the stress in a swift and comprehensive manner. One of the altered transcriptional regulators, CDIF630erm_01543, was found with increased amounts after fidaxomicin stress. This protein can be assigned to the GntR-family, whose members fulfil various biological functions in diverse bacterial groups. In *Mycobacterium spec*. it is known that GntR-family proteins are involved in resistance to antimicrobial compounds. In detail, overexpression of the GntR-family protein Rv1152 from *M. tuberculosis* results in higher resistance towards vancomycin [50]. In *M. smegmatis* overexpression of the GntR-family protein Ms0535 leads to enhanced resistance towards isoniazid, whose metabolite inhibits cell wall synthesis [51]. Additionally, the data obtained in this study may suggest a putative role of GntR-family proteins in the detoxification of the transcriptional inhibitor fidaxomicin.

The other transcriptional regulator altered in *C. difficile* in response to fidaxomicin stress is the MarR-homologue encoded by CDIF630erm_00601, whose amount was significantly decreased after treatment. In *E. coli*, MarR is a repressor, which regulates the expression of a regulon (Mar-regulon). The expression of the Mar-regulon confers resistance to organic solvents [52,53], oxidative stress agents [54], disinfectants [55,56], and multiple antibiotics [57,58]. Indeed, *mar* mutants showed increased susceptibility to inhibitors of gyrase, topoisomerase, transcription, translation, and cell wall synthesis. Besides repression of the *marABC* operon MarR also regulates other targets in *E. coli*, namely *arcABC*, *tolC*, *ompF*, *zwf*, *fumC*, *soxS*, and *inaA* [59]. Although there are homologues of those targets in *C. difficile*, only one, the ArcA-homologue CDIF630erm_01980, could be identified in the dataset presented here. Indeed, the amount of CDIF630erm_01980 increased more than two-fold after fidaxomicin treatment. However, this regulation was not statistically significant. As mutations in *E. coli*-MarR result in overexpression of ArcAB causing enhanced multidrug-efflux via TolC and therewith resistance to antimicrobials, it is tempting to speculate that the same effects can be observed in *C. difficile* and that the application of efflux pumps inhibitors in order to improve the activity of antibiotics (as suggested in Reference [60]) would be a treatment option.

## 5. Conclusions

The increasing incidence of *C. difficile* infection and the occurrence and spread of antibiotic-resistant strains emphasize the need for a detailed knowledge on the mode of action of antibiotics against this pathogen. This knowledge is necessary to understand resistance development and to develop new antimicrobial compounds to treat severe and potentially recurrent *C. difficile* infections. One way to contribute to this goal is the compilation of antibiotic signatures. Quantitative data for 425 *C. difficile* proteins obtained in this study point at very distinct proteomic adaptations of this important health-care associated pathogen to the antibiotics *C. difficile* infections are commonly treated with. Moreover, the derived proteomic signatures illustrate that the regulation of protein biosynthesis, DNA replication and repair, as well as the adaptation of the cell envelope are central elements of antibiotics adaptation. The availability of protein signature libraries in *C. difficile* will not only enhance the knowledge on different mechanisms of action, but will also provide a platform that supports the development and evaluation of new antibiotics.

## Figures and Tables

**Table 1:** This table lists all proteins which were significantly altered (*p*-value < 0.05, average log_2_ fold change >|0.8|) in either synthesis or amount after treatment of *C. difficile* 630∆*erm* with sublethal concentrations of vancomycin (VAN), metronidazole (MTZ) or fidaxomicin (FDX), respectively. A protein was assumed to be essential when it showed reciprocal best hits to its homologue in *C. difficile* R20291 after an analysis with Proteinortho [33].

Accession Number	Protein Name	R20291-Homologue	Function	Significantly Changed (*p* < 0.05, log_2_ Fold Change >|0.8|)					
				Amount			Synthesis		
				VAN	MTZ	FDX	VAN	MTZ	FDX
CDIF630erm_01408	RimM	CDR20291_1095	16S rRNA processing protein RimM	x	x			x	
CDIF630erm_03522	DapB1	CDR20291_3086	4-hydroxy-tetrahydrodipicolinate reductase		x	x		x	x
CDIF630erm_03707	MurB	CDR20291_3224	UDP-*N*-acetylenolpyruvoyl-glucosamine reductase		x	x			
CDIF630erm_02841	RpoZ	CDR20291_2474	DNA-directed RNA polymerase subunit omega		x	x			
CDIF630erm_01509	CDIF630erm_01509	CDR20291_1195	putative pyridoxine kinase		x				
CDIF630erm_02877	CDIF630erm_02877	CDR20291_2507	putative pyridoxal 5′-phosphate-dependent enzyme		x				
CDIF630erm_02708	GrpE	CDR20291_2355	stress protein (HSP-70 cofactor)		x				
CDIF630erm_04005	MnmG	CDR20291_3535	tRNA uridine 5-carboxymethyl-aminomethyl modification enzyme		x				
CDIF630erm_00239	MurA	CDR20291_0122	UDP-*N*-acetylglucosamine 1-carboxyvinyltransferase		x				
CDIF630erm_03828	Prs	CDR20291_3351	ribose-phosphate pyrophosphokinase		x				
CDIF630erm_00260	SecA1	CDR20291_0142	protein translocase subunit SecA1		x				
CDIF630erm_01292	MreB2	CDR20291_0982	rod shape-determining protein MreB			x			x
CDIF630erm_03832	MurC	CDR20291_3355	UDP-*N*-acetylmuramate-l-alanine ligase			x		x	
CDIF630erm_03585	Pgi	CDR20291_3146	glucose-6-phosphate isomerase				x		
CDIF630erm_02574	GuaB	CDR20291_2224	IMP dehydrogenase					x	
CDIF630erm_03992	RpsF	CDR20291_3523	30S ribosomal protein S6					x	
CDIF630erm_00160	Adk	CDR20291_0088	adenylate kinase						x
CDIF630erm_02772	LeuS	CDR20291_2410	leucine-tRNA ligas						x
CDIF630erm_03872	LysS	CDR20291_3389	lysine-tRNA ligase						x
CDIF630erm_00461	CDIF630erm_00461	CDR20291_0338	cobalt-dependent inorganic pyrophosphatase						x

**Table 2:** This table lists all proteins uniquely regulated after treatment with the antibiotic they were assigned to (vancomycin (VAN), metronidazole (MTZ), fidaxomicin (FDX)). Column “regulation” indicates up (↑) or downregulation (↓) in both sampling points (10 and 30 min) or changing regulation during adaptation (↑↓).

Antibiotic	Accession Number	Protein Name	Function	Regulation
VAN	CDIF630erm_00928	FloX	putative flavodoxin	↑
VAN	CDIF630erm_00005	GyrB	DNA gyrase subunit B	↑
VAN	CDIF630erm_01147	NagB	glucosamine-6-phosphate deaminase	↑
VAN	CDIF630erm_03585	Pgi	glucose-6-phosphate isomeras	↑
VAN	CDIF630erm_02205	CspC1	major cold shock protein CspC	↓
VAN	CDIF630erm_03462	Eno	enolase	↓
VAN	CDIF630erm_01010	SpeE	spermidine synthase	↓
VAN	CDIF630erm_03837	RsmA	ribosomal RNA small subunit methyltransferase A	↑↓
MTZ	CDIF630erm_03239	AtpA1	V-type ATP synthase subunit A	↑
MTZ	CDIF630erm_03421	BglA5	6-phospho-beta-glucosidase	↑
MTZ	CDIF630erm_03910	CarB1	carbamoyl-phosphate synthase large subunit	↑
MTZ	CDIF630erm_03912	CarB2	carbamoyl-phosphate synthase large subunit	↑
MTZ	CDIF630erm_00291	CDIF630erm_00291	ABC-type transport system, ATP-binding protein	↑
MTZ	CDIF630erm_02352	CDIF630erm_02352	putative multiprotein-complex assembly TPR repeat-containing protein	↑
MTZ	CDIF630erm_03387	CDIF630erm_03387	PTS system, glucose-like IIA component	↑
MTZ	CDIF630erm_03611	CDIF630erm_03611	putative D-isomer specific 2-hydroxyacid dehydrogenase	↑
MTZ	CDIF630erm_03703	CDIF630erm_03703	putative hydrolase, NUDIX family	↑
MTZ	CDIF630erm_03788	ComEB2	deoxycytidylate deaminase	↑
MTZ	CDIF630erm_01952	Def1	peptide deformylase	↑
MTZ	CDIF630erm_02427	FtnA	bacterial non-heme ferritin	↑
MTZ	CDIF630erm_03003	HisS	histidine-tRNA ligase	↑
MTZ	CDIF630erm_01131	Hpr	hydroxypyruvate reductase	↑
MTZ	CDIF630erm_02713	LepA	elongation factor 4	↑
MTZ	CDIF630erm_02845	LtaE	low specificity l-threonine aldolase	↑
MTZ	CDIF630erm_04005	MnmG	tRNA uridine 5-carboxymethylaminomethyl modification enzyme	↑
MTZ	CDIF630erm_01275	PolA	DNA polymerase I (POLI)	↑
MTZ	CDIF630erm_01160	PotA	ABC-type transport system, spermidine/putrescine ATP-binding protein	↑
MTZ	CDIF630erm_00260	SecA1	protein translocase subunit SecA1	↑
MTZ	CDIF630erm_01481	SelU	tRNA 2-selenouridine synthase	↑
MTZ	CDIF630erm_01811	TerD1	tellurium resistance protein TerD	↑
MTZ	CDIF630erm_02559	Tkt’1	transketolase, C-terminal section	↑
MTZ	CDIF630erm_00773	ToxB	toxin B	↑
MTZ	CDIF630erm_01943	YjiM	putative 2-hydroxyacyl-CoA dehydratase	↑
MTZ	CDIF630erm_01323	AckA	acetate kinase	↓
MTZ	CDIF630erm_02495	AmpP	xaa-pro aminopeptidase	↓
MTZ	CDIF630erm_02343	BipA	GTP-binding protein BipA	↓
MTZ	CDIF630erm_01509	CDIF630erm_01509	putative pyridoxine kinase	↓
MTZ	CDIF630erm_01961	CDIF630erm_01961	putative phenylalanyl-tRNA synthetase beta chain	↓
MTZ	CDIF630erm_02609	CDIF630erm_02609	DsrEFH-like protein	↓
MTZ	CDIF630erm_02799	CDIF630erm_02799	hypothetical protein	↓
MTZ	CDIF630erm_02825	CDIF630erm_02825	putative nitroreductase-like oxidoreductase	↓
MTZ	CDIF630erm_02877	CDIF630erm_02877	putative pyridoxal 5′-phosphate-dependent enzyme	↓
MTZ	CDIF630erm_02881	CDIF630erm_02881	ACT domain-containing protein	↓
MTZ	CDIF630erm_03134	CDIF630erm_03134	putative modulator of DNA gyrase, peptidase U62	↓
MTZ	CDIF630erm_03311	CDIF630erm_03311	PTS system, arabinose-specific IIA component	↓
MTZ	CDIF630erm_02708	GrpE	protein (HSP-70 cofactor)	↓
MTZ	CDIF630erm_02574	GuaB	IMP dehydrogenase	↓
MTZ	CDIF630erm_03527	Hpt2	hypoxanthine phosphoribosyltransferase	↓
MTZ	CDIF630erm_00239	MurA	UDP-N-acetylglucosamine 1-carboxyvinyltransferase	↓
MTZ	CDIF630erm_00113	ProS2	proline-tRNA ligase	↓
MTZ	CDIF630erm_03828	Prs	ribose-phosphate pyrophosphokinase	↓
MTZ	CDIF630erm_03992	RpsF	30S ribosomal protein S6	↓
MTZ	CDIF630erm_00009	RsbV	anti-sigma factor antagonist	↓
MTZ	CDIF630erm_03996	YedF	selenium metabolism protein YedF	↓
MTZ	CDIF630erm_01998	TerD4	tellurium resistance protein TerD	↑↓
FDX	CDIF630erm_00843	AcsE	5-methyltetrahydrofolate|corrinoid/iron-sulfur protein co-methyltransferase	↑
FDX	CDIF630erm_03250	AdhE2	aldehyde-alcohol dehydrogenase	↑
FDX	CDIF630erm_00160	Adk	adenylate kinase	↑
FDX	CDIF630erm_00657	ArcB	Delta (1)-pyrroline-2-carboxylate reductase	↑
FDX	CDIF630erm_00363	CDIF630erm_00363	phosphothreonine phosphatase	↑
FDX	CDIF630erm_00461	CDIF630erm_00461	cobalt-dependent inorganic pyrophosphatase	↑
FDX	CDIF630erm_01443	CDIF630erm_01443	ribonuclease J family protein	↑
FDX	CDIF630erm_01543	CDIF630erm_01543	transcriptional regulator, GntR family	↑
FDX	CDIF630erm_01816	CDIF630erm_01816	putative tellurite associated resistance protein	↑
FDX	CDIF630erm_01906	CDIF630erm_01906	uncharacterized protein	↑
FDX	CDIF630erm_02819	CDIF630erm_02819	PTS system, mannosylglycerate-specific IIA component	↑
FDX	CDIF630erm_03499	CDIF630erm_03499	putative nitroreductase-like oxidoreductase	↑
FDX	CDIF630erm_03853	CDIF630erm_03853	putative deoxyribonuclease	↑
FDX	CDIF630erm_00963	Cwp25	putative cell wall-binding protein	↑
FDX	CDIF630erm_01328	FabH	beta-ketoacyl-[acyl-carrier-protein] synthase 3	↑
FDX	CDIF630erm_00361	FliC	flagellin C	↑
FDX	CDIF630erm_00114	GltX	glutamate-tRNA ligase	↑
FDX	CDIF630erm_02400	Hcp	hydroxylamine reductase	↑
FDX	CDIF630erm_03727	HemB	delta-aminolevulinic acid dehydratase	↑
FDX	CDIF630erm_02772	LeuS	leucine-tRNA ligase	↑
FDX	CDIF630erm_03872	LysS	lysine-tRNA ligase	↑
FDX	CDIF630erm_03918	MetH	methionine synthase	↑
FDX	CDIF630erm_01292	MreB2	rod shape-determining protein MreB	↑
FDX	CDIF630erm_01369	NudF	ADP-ribose pyrophosphatase	↑
FDX	CDIF630erm_00345	PurH	bifunctional phosphoribosylaminoimidazolecarboxamide formyltransferase/IMP cyclohydrolase	↑
FDX	CDIF630erm_02743	SelD	selenide, water dikinase	↑
FDX	CDIF630erm_02763	TdcB	threonine dehydratase	↑
FDX	CDIF630erm_02348	ThrB	homoserine kinase	↑
FDX	CDIF630erm_03374	TreA	trehalose-6-phosphate hydrolase	↑
FDX	CDIF630erm_00231	VorB1	3-methyl-2-oxobutanoate dehydrogenase (ferredoxin), beta subunit	↑
FDX	CDIF630erm_02749	YcsE	putative 5-amino-6-(5-phospho-d-ribitylamino) uracil phosphatase	↑
FDX	CDIF630erm_03774	Alr	alanine racemase	↓
FDX	CDIF630erm_01216	ApeA	aminopeptidase ApeA	↓
FDX	CDIF630erm_00601	CDIF630erm_00601	transcriptional regulator, MarR family	↓
FDX	CDIF630erm_00852	CDIF630erm_00852	uncharacterized protein	↓
FDX	CDIF630erm_01899	CDIF630erm_01899	uncharacterized protein, UPF0145 family	↓
FDX	CDIF630erm_02209	CDIF630erm_02209	uncharacterized protein, AhpD-like	↓
FDX	CDIF630erm_02775	CDIF630erm_02775	putative hydrolase	↓
FDX	CDIF630erm_00360	FliT	flagellar protein FliT	↓

## Supplementary Materials

The following are available online at http://www.mdpi.com/2073-4409/7/11/213/s1, Table S1: All data on protein amounts, Table S2: All data on protein synthesis.

## References

[1] Lawson P.A., Citron D.M., Tyrrell K.L., Finegold S.M. (2016). Reclassification of *Clostridium difficile* as *Clostridioides difficile* (Hall and O’Toole 1935) Prévot 1938. Anaerobe.

[2] Mergenhagen K.A., Wojciechowski A.L., Paladino J.A. (2014). A review of the economics of treating *Clostridium difficile* infection. Pharmacoeconomics.

[3] Wilcox M.H., Howe R. (1995). Diarrhoea caused by *Clostridium difficile:* Response time for treatment with metronidazole and vancomycin. J. Antimicrob. Chemother..

[4] Gerding D.N. (2000). Treatment of *Clostridium difficile*-associated diarrhea and colitis. Curr. Top. Microbiol. Immunol..

[5] Surawicz C.M., Brandt L.J., Binion D.G., Ananthakrishnan A.N., Curry S.R., Gilligan P.H., McFarland L.V., Mellow M., Zuckerbraun B.S. (2013). Guidelines for diagnosis, treatment, and prevention of *Clostridium difficile* infections. Am. J. Gastroenterol..

[6] Perkins H.R. (1969). Specificity of combination between mucopeptide precursors and vancomycin or ristocetin. Biochem. J..

[7] Gerding D.N. (1997). Is there a relationship between vancomycin-resistant enterococcal infection and *Clostridium difficile* infection?. Clin. Infect. Dis..

[8] Gajdács M., Spengler G., Urbán E. (2017). Identification and antimicrobial susceptibility testing of anaerobic bacteria: Rubik’s cube of clinical microbiology?. Antibiotics.

[9] Miller W.R., Murray B.E., Rice L.B., Arias C.A. (2016). Vancomycin-resistant enterococci: Therapeutic challenges in the 21st century. Infect. Dis. Clin. N. Am..

[10] Barbut F., Decré D., Burghoffer B., Lesage D., Delisle F., Lalande V., Delmée M., Avesani V., Sano N., Coudert C. (1999). Antimicrobial susceptibilities and serogroups of clinical strains of *Clostridium difficile* isolated in France in 1991 and 1997. Antimicrob. Agents Chemother..

[11] Peláez T., Alcalá L., Alonso R., Rodríguez-Créixems M., García-Lechuz J.M., Bouza E. (2002). Reassessment of *Clostridium difficile* susceptibility to metronidazole and vancomycin. Antimicrob. Agents Chemother..

[12] Wong S.S.-Y., Woo P.C.-Y., Luk W.-K., Yuen K.-Y. (1999). Susceptibility testing of *Clostridium difficile* against metronidazole and vancomycin by disk diffusion and Etest. Diagn. Microbiol. Infect. Dis..

[13] Brazier J.S., Fawley W., Freeman J., Wilcox M.H. (2001). Reduced susceptibility of *Clostridium difficile* to metronidazole. J. Antimicrob. Chemother..

[14] Kuriyama A., Jackson J.L., Doi A., Kamiya T. (2011). Metronidazole-induced central nervous system toxicity: A systematic review. Clin. Neuropharmacol..

[15] Khanna S., Pardi D.S. (2012). *Clostridium difficile* infection: New insights into management. Mayo Clin. Proc..

[16] Louie T.J., Emery J., Krulicki W., Byrne B., Mah M. (2009). Opt-80 eliminates *Clostridium difficile* and is sparing of *Bacteroides* species during treatment of *C. difficile* infection. Antimicrob. Agents Chemother..

[17] Shue Y.K., Sears P.S., Shangle S., Walsh R.B., Lee C., Gorbach S.L., Okumu F., Preston R.A. (2008). Safety, tolerance, and pharmacokinetic studies of Opt-80 in healthy volunteers following single and multiple oral doses. Antimicrob. Agents Chemother..

[18] Artsimovitch I., Seddon J., Sears P. (2012). Fidaxomicin is an inhibitor of the initiation of bacterial RNA synthesis. Clin. Infect. Dis. Off. Publ. Infect. Dis. Soc. Am..

[19] Cornely O.A., Nathwani D., Ivanescu C., Odufowora-Sita O., Retsa P., Odeyemi I.A.O. (2014). Clinical efficacy of fidaxomicin compared with vancomycin and metronidazole in *Clostridium difficile* infections: A meta-analysis and indirect treatment comparison. J. Antimicrob. Chemother..

[20] Louie T.J., Cannon K., Byrne B., Emery J., Ward L., Eyben M., Krulicki W. (2012). Fidaxomicin preserves the intestinal microbiome during and after treatment of *Clostridium difficile* infection (CDI) and reduces both toxin reexpression and recurrence of CDI. Clin. Infect. Dis. Off. Publ. Infect. Dis. Soc. Am..

[21] Wösten M.M. (1998). Eubacterial sigma-factors. FEMS Microbiol. Rev..

[22] Freeman J., Wilcox M.H. (2001). Antibiotic activity against genotypically distinct and indistinguishable *Clostridium difficile* isolates. J. Antimicrob. Chemother..

[23] Fuchs S., Zühlke D., Pané-Farré J., Kusch H., Wolf C., Reiß S., Binh L.T.N., Albrecht D., Riedel K., Hecker M. (2013). Aureolib—A proteome signature library: Towards an understanding of *Staphylococcus aureus* pathophysiology. PLoS ONE.

[24] Hecker M., Antelmann H., Büttner K., Bernhardt J. (2008). Gel-based proteomics of Gram-positive bacteria: A powerful tool to address physiological questions. Proteomics.

[25] Bandow J.E., Brötz H., Leichert L.I.O., Labischinski H., Hecker M. (2003). Proteomic approach to understanding antibiotic action. Antimicrob. Agents Chemother..

[26] Hussain H.A., Roberts A.P., Mullany P. (2005). Generation of an erythromycin-sensitive derivative of *Clostridium difficile* strain 630 (630Δ*erm*) and demonstration that the conjugative transposon Tn*916*ΔE enters the genome of this strain at multiple sites. J. Med. Microbiol..

[27] Bernhardt J., Weibezahn J., Scharf C., Hecker M. (2003). *Bacillus subtilis* during feast and famine: Visualization of the overall regulation of protein synthesis during glucose starvation by proteome analysis. Genome Res..

[28] Bradford M.M. (1976). A rapid and sensitive method for the quantitation of microgram quantities of protein utilizing the principle of protein-dye binding. Anal. Biochem..

[29] Schwarz K., Fiedler T., Fischer R.-J., Bahl H. (2007). A Standard Operating Procedure (SOP) for the preparation of intra- and extracellular proteins of *Clostridium acetobutylicum* for proteome analysis. J. Microbiol. Methods.

[30] Moche M., Albrecht D., Maaß S., Hecker M., Westermeier R., Büttner K. (2013). The new horizon in 2D electrophoresis-new technology to increase resolution and sensitivity. Electrophoresis.

[31] Dannheim H., Riedel T., Neumann-Schaal M., Bunk B., Schober I., Spröer C., Chibani C.M., Gronow S., Liesegang H., Overmann J. (2017). Manual curation and reannotation of the genomes of *Clostridium difficile* 630Δ*erm* and *Clostridium difficile* 630. J. Med. Microbiol..

[32] Dembek M., Barquist L., Boinett C.J., Cain A.K., Mayho M., Lawley T.D., Fairweather N.F., Fagan R.P. (2015). High-throughput analysis of gene essentiality and sporulation in *Clostridium difficile*. mBio.

[33] Lechner M., Findeiß S., Steiner L., Marz M., Stadler P.F., Prohaska S.J. (2011). Proteinortho: Detection of (Co-)orthologs in large-scale analysis. BMC Bioinform..

[34] Wenzel M., Bandow J.E. (2011). Proteomic signatures in antibiotic research. Proteomics.

[35] Goldstein E.J.C., Citron D.M., Tyrrell K.L., Warren Y.A. (2010). Bactericidal activity of telavancin, vancomycin and metronidazole against *Clostridium difficile*. Anaerobe.

[36] Haft D.H., Selengut J.D., White O. (2003). The TIGRFAMs database of protein families. Nucleic Acids Res..

[37] Declerck P.J., De Ranter C.J. (1986). In vitro reductive activation of nitroimidazoles. Biochem. Pharmacol..

[38] Neumann-Schaal M., Hofmann J.D., Will S.E., Schomburg D. (2015). Time-resolved amino acid uptake of *Clostridium difficile 630*Δ*erm* and concomitant fermentation product and toxin formation. BMC Microbiol..

[39] Maaß S., Wachlin G., Bernhardt J., Eymann C., Fromion V., Riedel K., Becher D., Hecker M. (2014). Highly precise quantification of protein molecules per cell during stress and starvation responses in *Bacillus subtilis*. Mol. Cell. Proteomics.

[40] Hessling B., Bonn F., Otto A., Herbst F.-A., Rappen G.-M., Bernhardt J., Hecker M., Becher D. (2013). Global proteome analysis of vancomycin stress in *Staphylococcus aureus*. Int. J. Med. Microbiol..

[41] VanBogelen R.A., Neidhardt F.C. (1990). Ribosomes as sensors of heat and cold shock in *Escherichia coli*. Proc. Natl. Acad. Sci. USA.

[42] Chong P.M., Lynch T., McCorrister S., Kibsey P., Miller M., Gravel D., Westmacott G.R., Mulvey M.R. (2014). Canadian Nosocomial Infection Surveillance Program (CNISP). Proteomic analysis of a NAP1 *Clostridium difficile* clinical isolate resistant to metronidazole. PLoS ONE.

[43] Emerson J.E., Stabler R.A., Wren B.W., Fairweather N.F. (2008). Microarray analysis of the transcriptional responses of *Clostridium difficile* to environmental and antibiotic stress. J. Med. Microbiol..

[44] Steffens L.S., Nicholson S., Paul L.V., Nord C.E., Patrick S., Abratt V.R. (2010). *Bacteroides fragilis* RecA protein overexpression causes resistance to metronidazole. Res. Microbiol..

[45] Jeong J.Y., Mukhopadhyay A.K., Dailidiene D., Wang Y., Velapatiño B., Gilman R.H., Parkinson A.J., Nair G.B., Wong B.C., Lam S.K. (2000). Sequential inactivation of *rdxA* (HP0954) and *frxA*(HP0642) nitroreductase genes causes moderate and high-level metronidazole resistance in *Helicobacter pylori*. J. Bacteriol..

[46] Gal M., Brazier J.S. (2004). Metronidazole resistance in *Bacteroides* spp. carrying nim genes and the selection of slow-growing metronidazole-resistant mutants. J. Antimicrob. Chemother..

[47] Kwon D.H., Kato M., El-Zaatari F.A., Osato M.S., Graham D.Y. (2000). Frame-shift mutations in NAD(P)H flavin oxidoreductase encoding gene (*frxA*) from metronidazole resistant *Helicobacter pylori* ATCC43504 and its involvement in metronidazole resistance. FEMS Microbiol. Lett..

[48] Taylor D.E. (1999). Bacterial tellurite resistance. Trends Microbiol..

[49] Chasteen T.G., Fuentes D.E., Tantaleán J.C., Vásquez C.C. (2009). Tellurite: History, oxidative stress, and molecular mechanisms of resistance. FEMS Microbiol. Rev..

[50] Zeng J., Deng W., Yang W., Luo H., Duan X., Xie L., Li P., Wang R., Fu T., Abdalla A.E. (2016). *Mycobacterium tuberculosis* Rv1152 is a novel GntR family transcriptional regulator involved in intrinsic vancomycin resistance and is a potential vancomycin adjuvant target. Sci. Rep..

[51] Hu J., Zhao L., Yang M. (2015). A GntR family transcription factor positively regulates mycobacterial isoniazid resistance by controlling the expression of a putative permease. BMC Microbiol..

[52] White D.G., Goldman J.D., Demple B., Levy S.B. (1997). Role of the *acrAB* locus in organic solvent tolerance mediated by expression of *marA*, *soxS*, or *robA* in *Escherichia coli*. J. Bacteriol..

[53] Aono R. (1998). Improvement of organic solvent tolerance level of *Escherichia coli* by overexpression of stress-responsive genes. Extremophiles.

[54] Ariza R.R., Cohen S.P., Bachhawat N., Levy S.B., Demple B. (1994). Repressor mutations in the *marRAB* operon that activate oxidative stress genes and multiple antibiotic resistance in *Escherichia coli*. J. Bacteriol..

[55] Moken M.C., McMurry L.M., Levy S.B. (1997). Selection of multiple-antibiotic-resistant (mar) mutants of *Escherichia coli* by using the disinfectant pine oil: Roles of the *mar* and *acrAB* loci. Antimicrob. Agents Chemother..

[56] McMurry L.M., Oethinger M., Levy S.B. (1998). Overexpression of *marA*, *soxS*, or *acrAB* produces resistance to triclosan in laboratory and clinical strains of *Escherichia coli*. FEMS Microbiol. Lett..

[57] Okusu H., Ma D., Nikaido H. (1996). AcrAB efflux pump plays a major role in the antibiotic resistance phenotype of *Escherichia coli* multiple-antibiotic-resistance (Mar) mutants. J. Bacteriol..

[58] Goldman J.D., White D.G., Levy S.B. (1996). Multiple antibiotic resistance (*mar*) locus protects *Escherichia coli* from rapid cell killing by fluoroquinolones. Antimicrob. Agents Chemother..

[59] Alekshun M.N., Levy S.B. (1999). The mar regulon: Multiple resistance to antibiotics and other toxic chemicals. Trends Microbiol..

[60] Spengler G., Kincses A., Gajdács M., Amaral L. (2017). New roads leading to old destinations: Efflux pumps as targets to reverse multidrug resistance in bacteria. Molecules.

